# Hydrogen‐Bonding‐Directed Assembly of COF/Nanocluster Hybrids With Synergistic Roles for Efficient Solar Hydrogen Production

**DOI:** 10.1002/advs.76700

**Published:** 2026-07-27

**Authors:** Ailing Pan, De Yan, Anwu Xu, Hong Du, Abulikemu Abudu Rexit, Gang Zhang

**Affiliations:** ^1^ College of Chemistry and Chemical Engineering Xinjiang Normal University Urumqi People's Republic of China; ^2^ Department of Chemistry University of Science and Technology of China Hefei Anhui People's Republic of China; ^3^ Xinjiang Key Laboratory of Energy Storage and Photoelectrocatalytic Materials Urumqi People's Republic of China; ^4^ Xinjiang Joinworld Co., Ltd Urumqi People's Republic of China

**Keywords:** covalent organic frameworks, hydrogen‐bonding assembly, photocatalytic hydrogen evolution, photothermal effect, synergistic catalysis

## Abstract

Precise interfacial engineering and rational component synergy are critical for high‐performance photocatalysts but remain challenging to achieve. Here, we report a COF/nanocluster hybrid photocatalyst (NZT‐1) constructed by a hydrogen‐bonding‐directed assembly strategy. The catalyst comprises a photoactive TP‐TDS COF (a covalent organic framework built from 2,4,6‐triformylphloroglucinol and 3,7‐diaminodibenzo[b,d]thiophene‐5,5‐dioxide) and NiZnCo nanoclusters embedded in an N‐doped carbon matrix (NiZnCo‐NC). Unlike conventional physical mixing or random loading, this approach enables molecular‐level interfacial wiring between the COF and the nanoclusters. Within the ternary cocatalyst, a synergistic division of roles is inferred: Ni acts as a structural stabilizer, Zn fine‐tunes the Co d‐band center to optimize hydrogen adsorption, and Co serves as the active site. This architecture achieves a photocatalytic H_2_ evolution rate of 439.8 mmol g^−1^ h^−1^ and an apparent quantum yield of 18.6% at 500 nm—competitive with the best noble‐metal‐free systems. This work demonstrates that molecular‐level interfacial control combined with rational multi‐metal synergy provides a new paradigm for efficient, earth‐abundant artificial photosynthesis.

## Graphical abstract

Hydrogen‐bonding‐directed assembly enables molecular‐level integration of a covalent organic framework and NiZnCo nanoclusters. The engineered interface couples directional charge transfer, multimetal synergy, and photothermal‐assisted catalysis, providing a versatile strategy for efficient noble‐metal‐free solar hydrogen production.

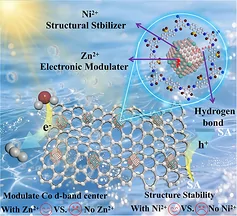

## 1 Introduction

Solar‐driven photocatalytic water splitting holds transformative potential for sustainable hydrogen production. However, practical implementation is hampered by the inherent trade‐offs among light absorption, charge separation, and catalytic kinetics in conventional semiconductor photocatalysts [1, 2, 3, 4]. Covalent organic frameworks (COFs) have emerged as a promising platform to address these challenges, owing to their designable porosity, tunable optoelectronic structures, and superior chemical stability [5, 6, 7]. Integrating photoactive linkers within a crystalline framework not only mitigates molecular aggregation but also promotes directional charge transport along π‐conjugated columns [8, 9, 10]. Despite these merits, the photocatalytic hydrogen evolution activity of pristine COFs is often limited by inefficient long‐range charge transport and the lack of directional electron flow toward catalytic sites, leading to severe bulk and interfacial recombination [11, 12].

Integrating cocatalysts, particularly earth‐abundant metal clusters, represents a mainstream strategy to create active interfaces that boost charge separation and provide catalytic sites [13, 14]. Encapsulating such clusters within N‐doped carbon (NC) matrices further enhances metal dispersion, stability, and electron conductivity [15, 16, 17, 18]. Recent studies hint at the potential of multi‐metal synergy; for instance, Ni can reinforce the NC framework while Zn modulates the electronic structure of active Co sites [19]. However, a critical and often overlooked bottleneck lies at the interface between the light‐harvesting COF and the catalytic cluster. Without precise control over interfacial bonding and energy alignment, electron transfer remains stochastic, and the potential synergy within multi‐metal cocatalysts cannot be fully leveraged [20, 21, 22]. Achieving directional charge transport and maximized host—cocatalyst synergy thus requires rational interface engineering beyond simple physical integration.

In this work, we construct a COF/NiZnCo nanocluster hybrid (NZT‐x) via hydrogen‐bonding‐directed assembly. Unlike physical mixing or wet impregnation, our approach creates molecular‐level interfacial wiring between TP‐TDS COF and NiZnCo embedded in an N‐doped carbon matrix. Hydrogen bonding has been used in COF photocatalysis for self‐assembly [23] or intralayer modulation [24], but not as a direct interfacial linker to a ternary metal nanocluster. Our work goes further by using a ternary NiZnCo nanocluster and, for the first time, deconvoluting the roles of each metal: Ni stabilizes the framework, Zn tunes the Co d‐band center, and Co acts as the active site (supported by ICP, STEM, XAFS, and DFT). The NZT‐1 hybrid achieves an H_2_ evolution rate of 439.8 mmol g^−1^ h^−1^ and an AQY of 18.6% at 500 nm. Although this is lower than our recent coordination‐based single‐atom system (Nat. Commun. 2026, 534.6 mmol g^−1^ h^−1^) [25], the design principle is distinct—hydrogen‐bonded ternary nanocluster assembly—and remains competitive among noble‐metal‐free systems of this type. Thus, precise interfacial engineering with rational multi‐metal synergy offers an alternative route for efficient, earth‐abundant photocatalysts.

## 2 Results and Discussion

Scheme 1 illustrates the hydrogen‐bonding‐directed assembly strategy central to constructing the NZT‐x architecture with a precisely engineered interface. The process begins with the pyrolysis of a NiZnCo‐ZIF‐67 precursor to yield conductive NiZnCo nanoclusters within an N‐doped carbon matrix (NiZnCo‐NC). These clusters then serve as active templates for the subsequent oriented nucleation and growth of the TP‐TDS COF via a three‐step process involving surface functionalization, interfacial nucleation, and directed growth. This strategy is designed to ensure intimate contact and molecular‐level interaction between the light‐harvesting COF and the catalytic metal sites, which we hypothesize is foundational to the observed performance.

**SCHEME 1:**
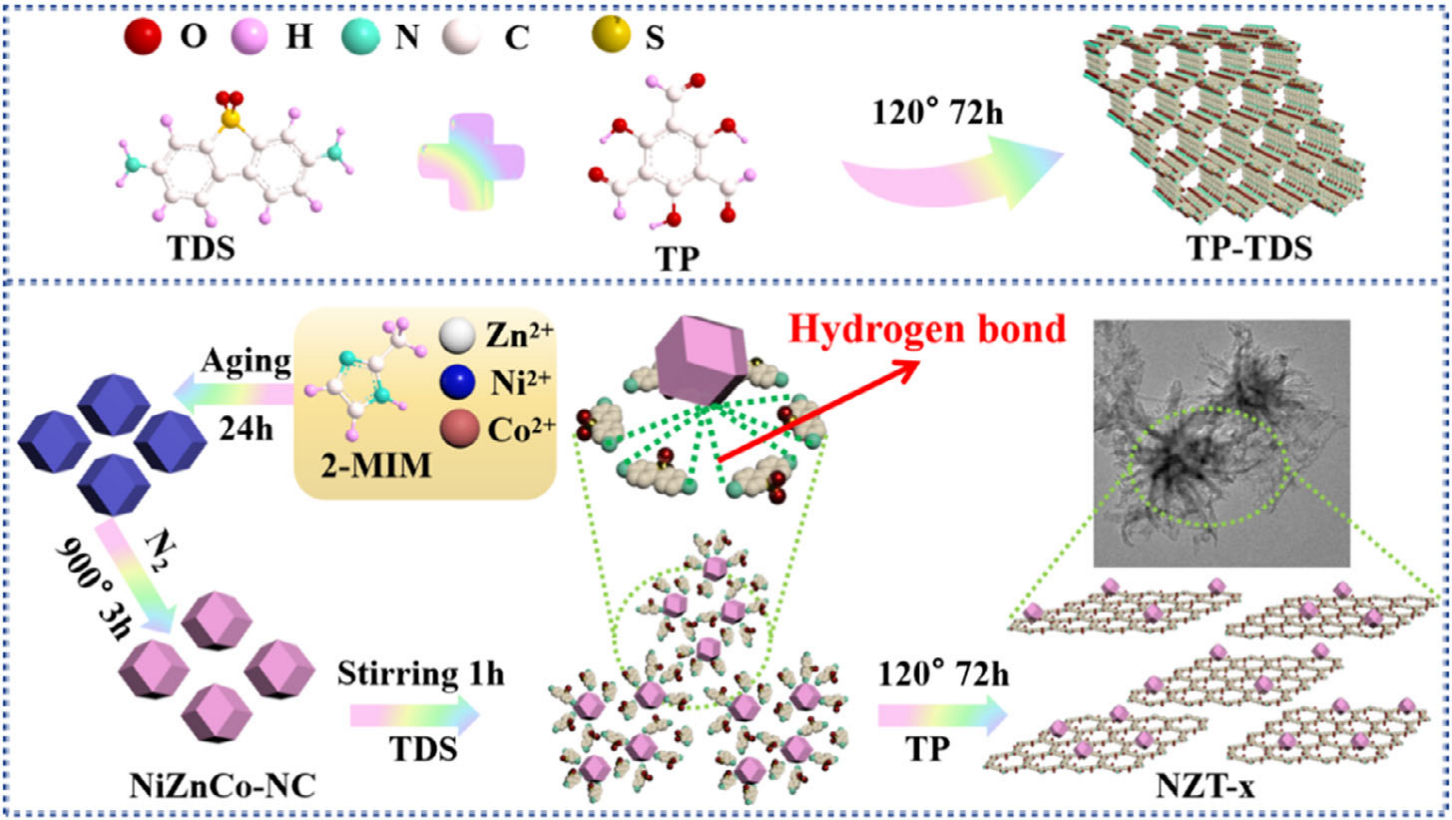
Schematic illustration of the synthetic procedures for NiZnCo‐NC, TP‐TDS COF, and NZT‐1.

The structural evolution from precursors to the final hybrid was characterized by X‐ray diffraction (XRD) and electron microscopy. XRD indicates the phase purity of the NiZnCo‐ZIF‐67 precursor (Figure S1) and its successful conversion to NiZnCo‐NC, evidenced by the emergence of graphitic carbon and metallic Co signatures (Figure S2) [26]. Specifically, a broad diffraction peak centered at approximately 26° can be indexed to the (002) plane of graphitic carbon, indicating carbonization and graphitization of the organic ligands from the ZIF precursor, while the peaks at 44°, 51°, and 76° correspond to metallic Co, confirming the reduction of Co species during pyrolysis. Meanwhile, high‐resolution transmission electron microscopy (HRTEM) (Figure 1g) reveals graphitic carbon shells continuously wrapping around metallic Co nanocrystals embedded within the carbon matrix. This core‐shell morphology, together with the absence of CoO or Co_3_O_4_ peaks in XRD, confirms that the product is the desired NiZnCo‐NC structure with metallic Co nanoclusters encapsulated in a graphitic carbon matrix. Critically, the XRD pattern of NZT‐1 (Figure S3) indicates the successful integration of both components. Although the characteristic diffraction peak of TP‐TDS COF is retained after hybridization, its intensity becomes weaker, and the peak broadens, indicating a partial reduction in long‐range ordering rather than complete loss of crystallinity. This behavior is attributed to the confined growth of the COF around the NiZnCo‐NC nanoclusters and the resulting perturbation of interlayer *π*–*π* stacking interactions [27]. Additionally, an acid stability test (Figure S4) definitively rules out unwashed precursors, suggesting that Ni and Zn are embedded within the stable NC framework and are not removed by acid washing.

**FIGURE 1:**
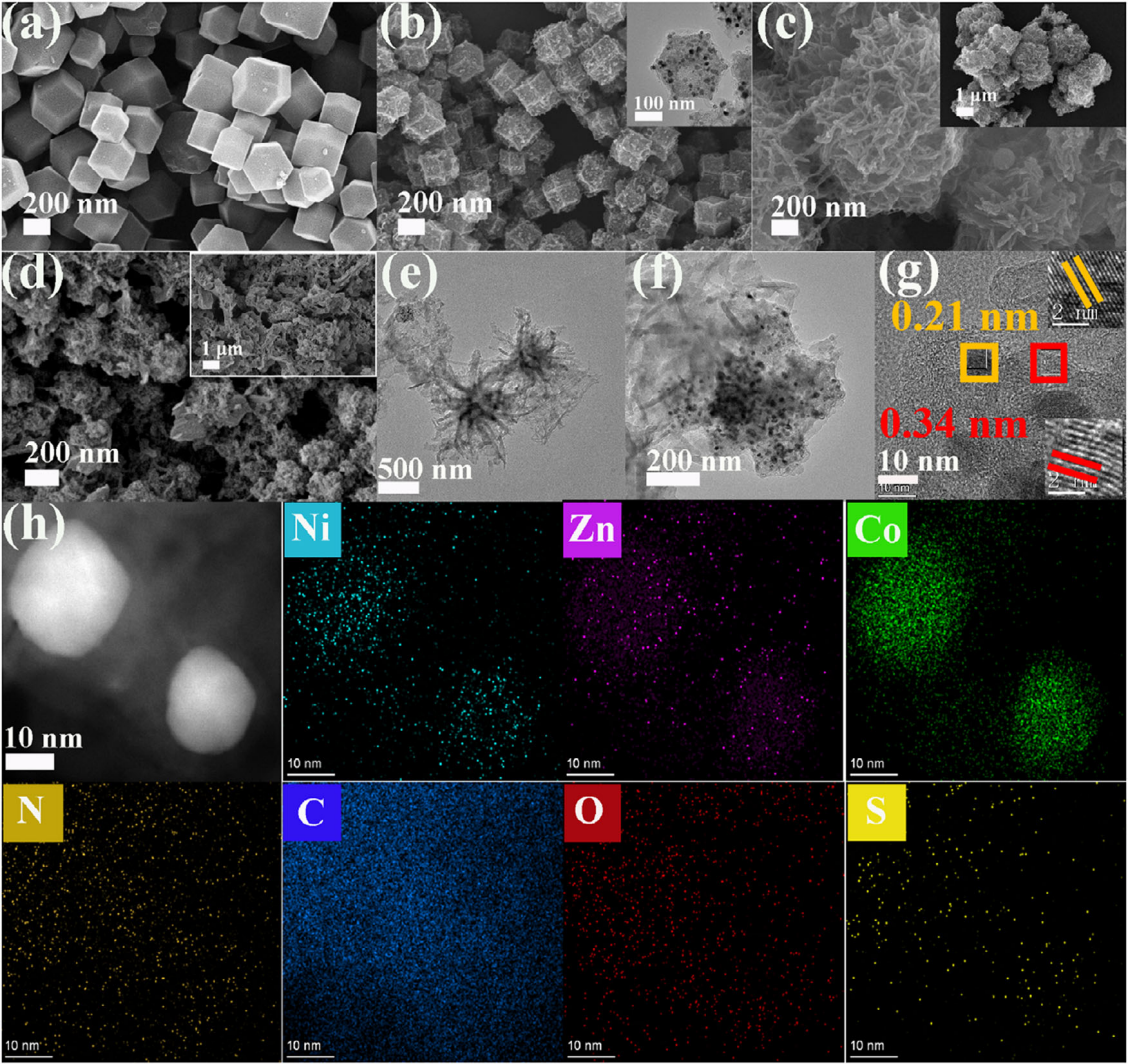
Morphological and structural characterization of the NZT‐1. (a–d) Scanning electron microscopy (SEM) images: (a) NiZnCo‐ZIF‐67 precursor, (b) NiZnCo‐NC, (c) TP‐TDS‐COF, and (d) the hybrid NZT‐1; (e–g) High‐resolution transmission electron microscopy (HRTEM) images and (h) HAADF‐STEM image of NZT‐1 and the corresponding elemental mappings.

Electron microscopy reveals the morphological hierarchy of this architecture. The NiZnCo‐ZIF‐67 dodecahedron (Figure 1a) transforms into a roughened but shape‐retained NiZnCo‐NC after pyrolysis, with HR‐TEM suggesting embedded crystalline metal nanoparticles (Figure 1b). In contrast, the pristine TP‐TDS COF forms chrysanthemum‐like spherical superstructures with open porosity (Figure 1c). The final NZT‐1 hybrid exhibits a uniform composite morphology (Figure 1d), where NiZnCo‐NC particles are seamlessly incorporated within a continuous COF matrix without agglomeration, a hallmark of the hydrogen‐bonding‐directed assembly. High‐resolution TEM of NZT‐1 (Figure 1e–g) provides nanoscale verification of the hybrid architecture. The images reveal metallic Co nanoparticles with a lattice spacing of 0.21 nm, corresponding to the (111) plane of fcc Co [28], surrounded by graphitic carbon shells with a lattice spacing of 0.34 nm, assigned to the (002) plane of graphite. To further clarify the structural relationship among Ni, Zn, and Co, high‐angle annular dark‐field scanning transmission electron microscopy (HAADF‐STEM) coupled with EDS elemental mapping was performed (Figure 1h). The elemental maps reveal that Ni, Zn, and Co are spatially co‐localized within the same nanocluster regions, showing highly overlapping distributions without detectable Ni‐rich or Zn‐rich segregated particles. Quantitative ICP‐MS analysis further reveals that Ni and Zn are present at only 0.025 and 0.055 wt.%, respectively, while Co accounts for about 15 wt.% (Table S1). These trace concentrations indicate that Ni and Zn are present at very low levels relative to Co. Combined with the absence of isolated Ni‐ or Zn‐rich particles observed by STEM‐EDS, this supports a model in which Ni and Zn are atomically dispersed on or near the surface of the Co nanoclusters, rather than forming separate phases. While the above results indicate the successful structural integration of TP‐TDS and NiZnCo‐NC, direct evidence for the molecular interaction responsible for such intimate coupling is still required. Therefore, FT–IR and solid‐state 1H NMR spectroscopies were employed to probe the proposed interfacial hydrogen‐bonding interaction.

FT–IR spectra of pristine TP‐TDS COF, pure NiZnCo‐NC, and the NZT‐x series (NZT‐0.5, NZT‐1, NZT‐2) are shown in Figure 2a. The characteristic imine (C═N) band of the COF at 1255 cm^−1^ appears in all NZT‐x composites, indicating that the COF framework remains intact after hybridization. The N─H stretching band of pristine COF is centered at ∼3698 cm^−1^. Upon incorporation of NiZnCo‐NC, this band progressively shifts to lower wavenumbers: to ∼3676 cm^−1^ for NZT‐0.5, ∼3642 cm^−1^ for NZT−1, and ∼3630 cm^−1^ for NZT‐2. The red shift increases with NiZnCo‐NC content, indicating that the local vibrational environment of the N─H groups is altered by interfacial interactions. Pure NiZnCo‐NC shows no N─H absorption in this region, ruling out spectral interference. These shifts provide qualitative evidence for N─H···N hydrogen bonding between the residual amine groups of the COF and N‐containing species on the NiZnCo‐NC surface. This interpretation is independently supported by solid‐state ^1^H NMR (Figures S7–S9), which shows a downfield shift of the N─H protons in NZT‐1, a well‐established signature of hydrogen bond formation [29, 30]. To further exclude simple physical mixing or weak adsorption, we prepared a control sample by manually grinding pre‐synthesized TP‐TDS COF and NiZnCo‐NC (milled NZT‐1). As shown in Figure S10, this physically mixed sample exhibits a photocatalytic H2 evolution rate nearly 50 times lower than that of the hydrogen‐bonded hybrid NZT‐1, indicating that the intimate, ordered interface created by hydrogen‐bonding‐directed assembly is essential for high activity. These hydrogen bonds weaken the interlayer *π*–*π* stacking of the COF, thereby promoting its exfoliation into ultrathin nanosheets. To statistically validate this morphological transformation, multiple atomic force microscopy (AFM) measurements were performed on randomly selected regions of both pristine TP‐TDS COF and NZT‐1 samples. The thickness distribution histograms (Figure S11), derived from dozens of individual flakes, show that the pristine COF predominantly exhibits thicknesses in the range of ∼50–75 nm, characteristic of compact bulk structures, whereas the NZT‐1 hybrid predominantly consists of ultrathin nanosheets with thicknesses concentrated around ∼2–5 nm (representative AFM images shown in Figure 2b,c). These results suggest that the dramatic thinning after hybridization is reproducible and not an artifact of isolated local regions. This exfoliation is attributed to the hydrogen‐bonding‐directed interfacial assembly, which perturbs regular interlayer stacking of the COF and promotes nanosheet formation during the growth process. This dramatic thinning, driven by the templating effect during directed growth, vastly increases exposed active sites and enhances light harvesting — structural attributes anticipated to directly boost photocatalytic performance [31]. Although the COF in NZT‐1 shows reduced long‐range stacking order compared to the pristine COF (Figure S11), the dramatic thinning to ∼2–5 nm shortens charge transport distances and increases active site exposure, more than compensating for any potential loss in crystallinity. To verify whether these structural advantages indeed translate into improved catalytic performance, photocatalytic hydrogen evolution measurements were subsequently conducted under simulated solar irradiation. The photocatalytic hydrogen evolution performance of NZT‐1 under simulated solar irradiation (AM 1.5G) is enhanced. It achieves a rate of 439.8 mmol g^−1^ h^−1^ (Figure 2d) (Video S1), representing a ∼1700‐fold enhancement over the pristine COF and surpassing a benchmark 1 wt.% Pt‐loaded COF (Figure S12). In addition, High activity is maintained under visible light (336.1 mmol g^−1^ h^−1^, AQY = 18.6% at 500 nm, Figure S14 and Table S2). The AQY remained stable under varying light intensities (16.7%–18.6% at 40–86 mW cm^−2^, Table S3), and the measurements were performed under continuous stirring with low catalyst loading to minimize mass‐transfer limitations. The solar‐to‐chemical conversion (SCC) efficiency of NZT‐1 under AM 1.5G irradiation was calculated to be 14.5% (see Supporting Information for details), further demonstrating its excellent solar‐to‐hydrogen conversion capability. This performance is optimized at a specific NiZnCo‐NC loading, positioning NZT‐1 among top‐performing noble‐metal‐free systems (Figure S16). Without a sacrificial agent (ascorbic acid), NZT‐1 shows negligible H_2_ evolution (Figure S12), suggesting that the sacrificial agent is essential for hole scavenging and efficient charge separation. To further understand the origin of the enhanced photocatalytic performance, water contact angle measurements were conducted to evaluate the surface wettability of the samples (Figure S17). The pristine TP‐TDS COF exhibits a contact angle of 68.2°, indicating moderate hydrophilicity. In contrast, NiZnCo‐NC alone is superhydrophilic with a contact angle below 5°, and the NZT‐1 hybrid shows a markedly reduced contact angle of 14.2° at 1 s, with the droplet rapidly spreading further within 2 s. This enhanced wettability is beneficial for promoting water adsorption, facilitating interfacial mass transfer, and improving the accessibility of catalytic active sites, thereby contributing to the efficient photocatalytic hydrogen evolution of NZT‐1 [32]. Operational stability was evaluated over ten cycles. The catalyst maintained robust performance for the first three cycles, after which a gradual decay was observed (Figure 2e). To clarify the origin of this activity decay, the spent NZT‐1 catalyst was examined by XRD, TEM, and XPS after photocatalytic cycling (Figures S18–S21). The XRD pattern of the used catalyst retains the characteristic diffraction peaks of TP‐TDS COF, with only slight broadening and attenuation, indicating that the COF framework is largely preserved without obvious structural collapse. TEM observations further reveal that the NiZnCo nanoclusters remain uniformly dispersed within the COF matrix, and no obvious nanoparticle aggregation or newly formed crystalline phases are detected. These results demonstrate that the overall hybrid architecture of NZT‐1 is well maintained during photocatalysis. Post‐reaction XPS identifies surface oxidation of metallic Co as the primary cause of activity loss [33]. The stable performance in the first three cycles suggests that the surface oxide layer forms gradually; once a critical thickness is reached, charge transfer becomes hindered and activity declines. Thus, Co surface oxidation, rather than structural collapse or aggregation, is the dominant deactivation mechanism. To probe the distinct and synergistic roles of Ni and Zn, we conducted a compositional deconstruction experiment. Control hybrid catalysts were fabricated by replacing the ternary NiZnCo‐NC component with analogues lacking key metals: CT‐1 (using Co‐NC, lacking both Ni and Zn), NCT‐1 (using NiCo‐NC, lacking Zn), and ZCT‐1 (using ZnCo‐NC, lacking Ni). Their photocatalytic activities under AM 1.5G reveal a non‐linear hierarchy culminating in NZT‐1's peak performance (Figure 2f). This trend is directly correlated with their structural integrity (Figure S22). Electron microscopy shows that Ni is essential for maintaining framework integrity: NiCo‐NC preserves the dodecahedral morphology, while ZnCo‐NC suffers complete collapse. This structural stabilization by Ni explains the superior activity of NCT‐1 over ZCT‐1. However, the extraordinary activity leap from the stable binary NCT‐1 to the ternary NZT‐1 — by a factor of ∼3.4 — signals a contribution beyond mere structural stability. We hypothesized that this leap originates from the electronic synergy enabled by Zn incorporation, a hypothesis that we subsequently test and indicate through density functional theory calculations.

**FIGURE 2:**
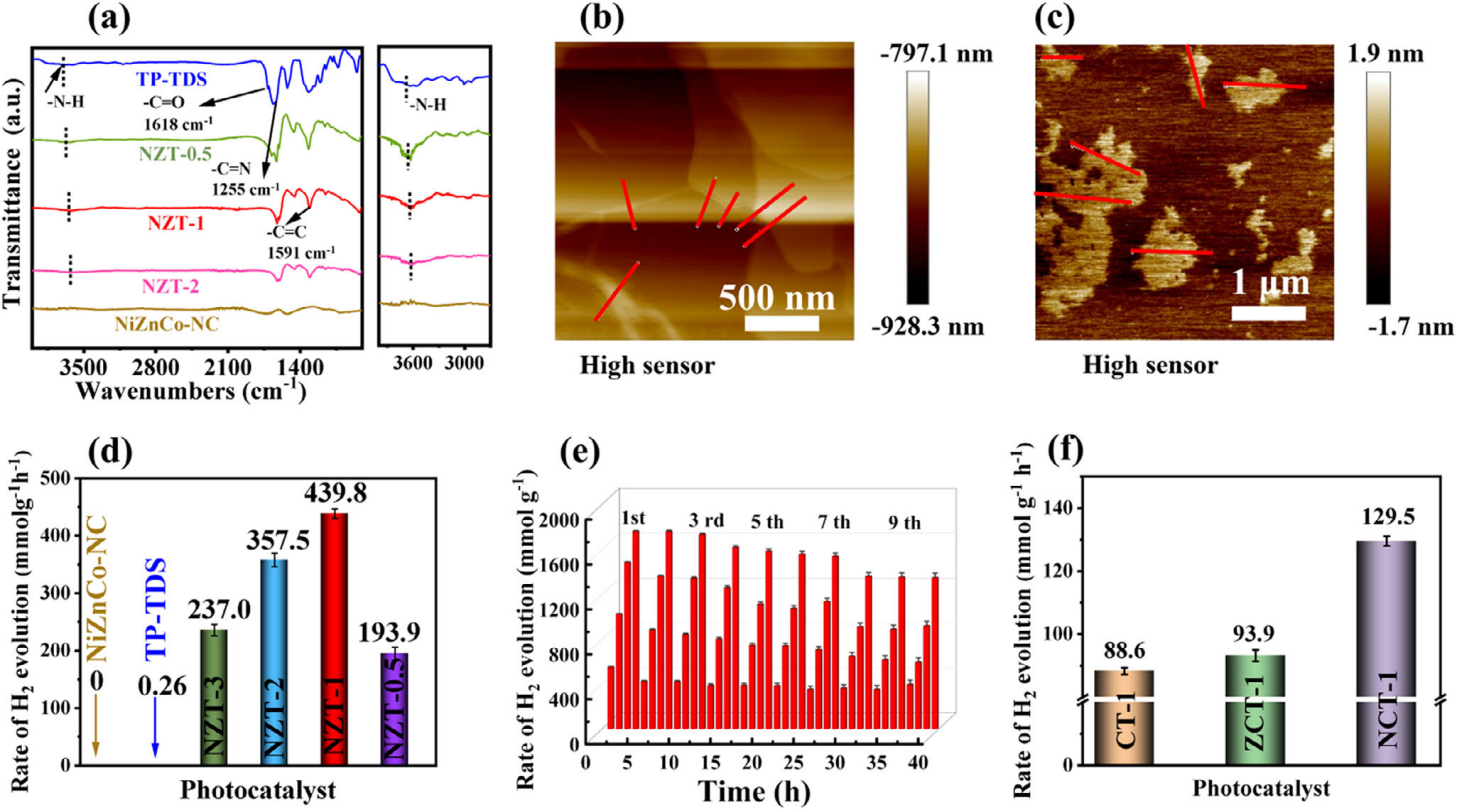
(a) FT—IR spectra of TP‐TDS COF, NiZnCo‐NC and NZT‐x hybrids; AFM images of (b) pristine TP‐TDS COF and (c) NZT‐1, confirming exfoliation into ultrathin nanosheets; (d) Photocatalytic H_2_ evolution rates under AM 1.5G; (e) Cycling stability test of the optimized NZT‐1 over ten consecutive runs; (f) Photocatalytic H_2_ evolution rates of different samples (all quantitative performance measurements were repeated at least three times independently (*n* = 3)).

To gain deeper insight into the origin of this performance enhancement and to distinguish the respective roles of Ni and Zn, density functional theory (DFT) calculations were performed. The calculated hydrogen adsorption free energy (ΔGH^*^) serves as a key descriptor for catalytic efficiency (Figure 3a). For pristine Co‐NC, ΔGH^*^ is excessively negative (−2.24 eV), indicating an overly strong H^*^ binding that would poison the active site and impede hydrogen desorption. Introducing either Ni or Zn mitigates this strong adsorption, yielding more moderate ΔGH^*^ values of −0.33 eV (NiCo‐NC) and −0.38 eV (ZnCo‐NC). Notably, the ternary NiZnCo‐NC achieves a near‐optimal ΔGH^*^ of +0.087 eV, suggesting its Co sites are close to the peak of the theoretical activity volcano for the hydrogen evolution reaction (HER) [19, 34]. This dramatic optimization is rooted in electronic structure modulation. Projected density of states (PDOS) analysis (Figure 3b–e) indicates a systematic downshift of the Co d‐band center upon doping: from −1.28 eV (Co‐NC) to −1.31 eV (NiCo‐NC), −1.40 eV (ZnCo‐NC), and −1.37 eV (NiZnCo‐NC). According to the d‐band model, this downshift reduces the energy and occupancy of the antibonding states in the Co─H bond, thereby weakening the overall bond strength. This electronic effect is reflected in the more positive (less negative) ΔGH^*^ values [35]. The results suggest that both Ni and Zn act as electronic modulators for Co, with Zn inducing the more pronounced d‐band shift and thus likely playing a dominant role in fine‐tuning the adsorption energy. In conjunction with the experimental evidence for Ni's role as a structural stabilizer, these computational results support a coherent synergistic division of roles within the ternary cocatalyst: Zn primarily serves as an electronic modulator to optimize the Co active sites, while Ni ensures the architectural integrity necessary to host them. This cooperation appears to be associated with the enhanced intrinsic activity of the NiZnCo‐NC component.

**FIGURE 3:**
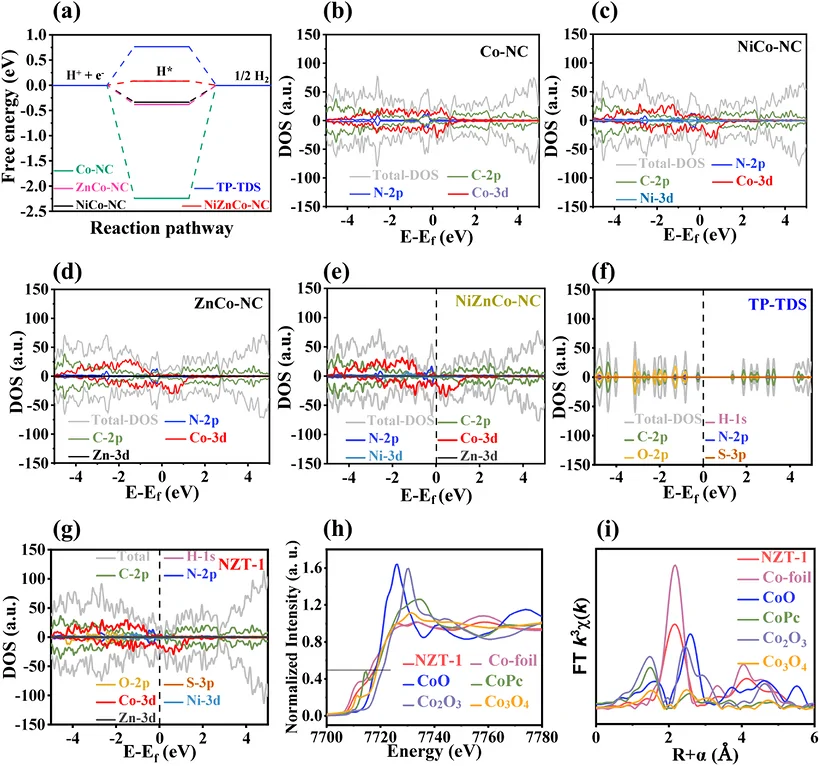
Electronic structure characterization and HER thermodynamic analysis. (a) Calculated Gibbs free energy profiles for HER on different samples; Density of states (DOS) for (b) Co‐NC; (c) NiCo‐NC; (d) ZnCo‐NC; (e) NiZnCo‐NC; (f) TP‐TDS; (g) the integrated NZT‐1 heterostructure; (h) Co K‐edge X‐ray absorption near‐edge structure (XANES) spectra; (i) Fourier transformation (FT) of the k^2^‐weighted extended X‐ray absorption fine structure (EXAFS) spectra at the Co K‐edge.

While these results explain the intrinsic catalytic superiority of NiZnCo‐NC, the overall photocatalytic performance of NZT‐1 also depends critically on the efficiency of interfacial charge separation and transport. Therefore, we next examined the electronic structure and charge‐transfer behavior of the complete hybrid system. DFT calculations (Figure 3e–g) indicate an advantageous electronic landscape: NiZnCo‐NC exhibits metallic character for rapid charge conduction, while the TP‐TDS COF possesses a suitable optical gap (DFT: 1.51 eV; experimental: 2.08 eV, Figure S33) [36]. The hybrid shows enhanced electronic states near the Fermi level, which may indicate strong interfacial electronic hybridization that could facilitate charge transfer across the junction. Such enhanced interfacial coupling is consistent with the improved charge‐separation behavior and is expected to promote electron transport from the COF to the cocatalyst. The primary catalytic sites within this architecture can be inferred by combining theory and experiment. Having established the favorable charge‐transfer characteristics of the heterojunction, we next sought to observe the catalytic sites responsible for hydrogen evolution. DFT calculations suggest metallic Co as the most active site for HER. This assignment is strongly supported by X‐ray absorption spectroscopy (XAS, Figure 3h,i). The Co K‐edge XANES spectrum of NZT‐1 closely resembles that of Co foil rather than oxidized Co references, confirming that Co predominantly exists in the metallic state after pyrolysis. The EXAFS spectra are dominated by a peak corresponding to metallic Co─Co coordination [37], with quantitative fitting indicating well‐defined cobalt nanoclusters as the predominant species, ruling out significant contributions from oxidized or nitrogen‐coordinated Co. Furthermore, no statistically meaningful Co─Ni or Co─Zn scattering paths were detected, consistent with the HAADF‐STEM‐EDS (Figure 1h) and ICP‐MS results (Table S1), indicating that Ni and Zn are not incorporated into the Co lattice but instead modulate the catalytic properties of neighboring Co sites through local interfacial interactions. Collectively, the XANES and EXAFS results support the conclusion that metallic Co nanoclusters serve as the primary HER active sites in the NZT‐1 system. In summary, these results elucidate that efficient charge dynamics and high activity require both the optimized electronic structure of the hybrid and the molecularly precise interface that integrates its components.

To elucidate the photophysical origins of the enhanced performance, we conducted a suite of optical and photoelectronic characterizations. UV–vis spectroscopy (Figure 4a) shows that NZT‐1 exhibits markedly enhanced and broadened light absorption across the UV–vis–NIR range compared to the pristine TP‐TDS COF. This enhancement is attributed to the synergistic contribution of the metallic NiZnCo‐NC component, which not only increases photon harvesting but acts as an efficient electron acceptor, providing a conductive pathway for rapid charge separation and transfer [25]. Crucially, the absorbed photons are converted into long‐lived charge carriers with high efficiency. Transient photocurrent response measurements (Figure 4b) show a significantly higher and more stable photocurrent density for NZT‐1, indicating superior charge separation and collection. EIS Nyquist plots (Figure S37) show that NZT‐1 exhibits a much smaller semicircle radius than TP‐TDS COF, indicating lower charge transfer resistance. This qualitative trend is consistent with the transient photocurrent and the time‐resolved photoluminescence (TR‐PL, Figure 4c), which reveals a substantial increase in the average charge carrier lifetime from 0.47 ns for the pristine COF to 3.32 ns for NZT‐1, providing direct evidence of inhibited charge carrier recombination. The higher photocurrent and prolonged carrier lifetime in NZT‐1 indicate that photogenerated electrons in the COF are efficiently transferred to the NiZnCo nanoclusters, where they participate in proton reduction. This assignment is further supported by the near‐optimal ΔGH^*^ calculated for Co sites (Figure 3a) and the absence of significant HER activity on the COF alone. The underlying reason for this enhanced charge separation is traced to a significant reduction in exciton binding energy. Temperature‐dependent PL spectroscopy (Figure 4d,e) determines the exciton binding energy of NZT‐1 to be 53.19 meV, which is 26% lower than that of the pristine COF (72.26 meV). This reduction, facilitated by the strong interfacial coupling and extended electronic conjugation within the hybrid, promotes the dissociation of photogenerated excitons into free charge carriers. This fundamental change in photophysics directly explains the observed prolonged carrier lifetimes and enhanced photocurrents. Furthermore, nitrogen physisorption measurements (Figure 4f) suggest that the architecture supports efficient mass transport. Although the specific surface area of NZT‐1 (410.83 m^2^ g^−1^) is lower than that of the COF (986.51 m^2^ g^−1^) due to pore occupation by the cocatalyst, the material retains a well‐defined mesoporous structure (pore size ∼8.24 nm, Figure S38), ensuring unhindered diffusion of reactants to the highly active sites [38, 39].

**FIGURE 4:**
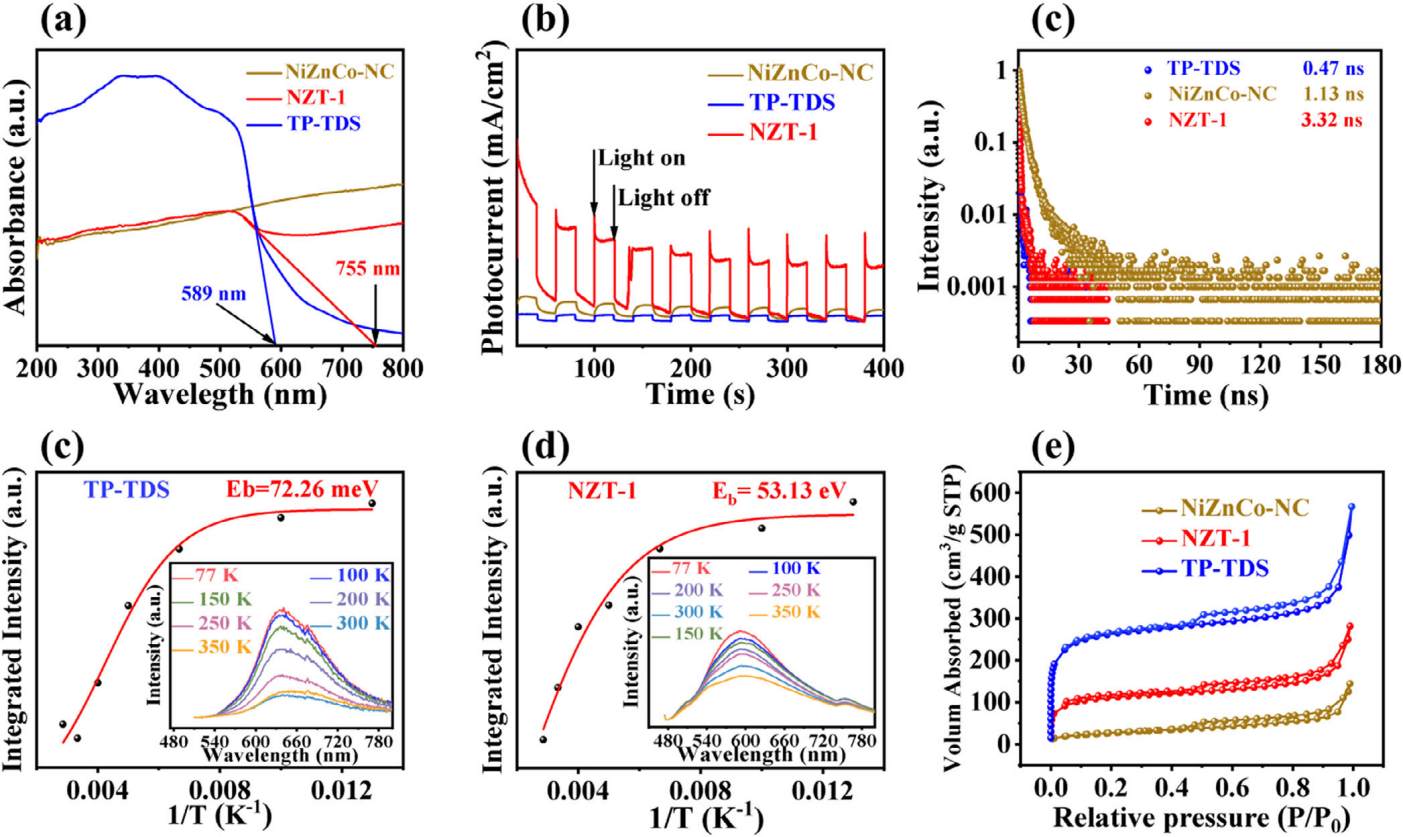
Photophysical and textural properties of the bioinspired photocatalytic architectures. (a) UV–vis absorption spectra; (b) Transient photocurrent responses, (c) Time‐resolved PL decay profiles of TP‐TDS COF, NiZnCo‐NC and NZT‐1 hybrids; Temperature‐dependent integrated PL emission intensity for (d) TP‐TDS COF and (e) NZT‐1, used for exciton binding energy determination; (f) N_2_ adsorption–desorption isotherm of TP‐TDS COF, NiZnCo‐NC and NZT‐1.

Although the above results demonstrate improved charge generation and separation, the driving force responsible for directional charge migration across the interface remains to be clarified. Building on the enhanced charge generation, we deciphered the interfacial charge transfer dynamics and built‐in electric field (IEF) formation. The process originates from an intrinsic electronic asymmetry: ultraviolet photoelectron spectroscopy (UPS) measurements (Figure S39) show NiZnCo‐NC has a lower work function (2.59 eV) than the TP‐TDS COF (2.96 eV). This gradient drives spontaneous electron transfer from NiZnCo‐NC to the COF upon contact, as indicated by dark‐state X‐ray photoelectron spectroscopy (XPS) (Figure 5a,b). This charge separation establishes a built‐in electric field (IEF) at the interface. DFT‐calculated differential charge density (Figure 5c) visualizes this coupled interface at the atomic scale, mapping the static charge redistribution (electron accumulation on COF, depletion on NiZnCo‐NC) that underlies the IEF. While efficient charge separation ensures rapid electron delivery to catalytic sites, achieving high hydrogen evolution activity also requires favorable surface reaction kinetics. Therefore, we further investigated whether a photothermal contribution exists in the NZT‐1 system [40]. In addition to the photo‐driven charge separation process, the localized photothermal effect plays an important role in enhancing the intrinsic reaction kinetics. The elevated local temperature generated under illumination accelerates surface proton reduction kinetics by facilitating hydrogen adsorption/desorption processes and lowering the activation barrier for hydrogen evolution. DFT calculations further reveal that increasing the temperature shifts the ΔGH^*^ value of NiZnCo‐NC from +0.087 to +0.150 eV (Figure 5d), indicating that elevated temperature facilitates H^*^desorption kinetics and accelerates interfacial proton reduction. Therefore, while the IEF is primarily responsible for directional charge separation and electron transport, the photothermal effect mainly improves the intrinsic catalytic kinetics after charge transfer has occurred. In addition, local heating may increase carrier mobility and interfacial charge‐transfer rates, further contributing to the overall photocatalytic performance. To experimentally verify the existence of such a photothermal contribution, infrared thermography measurements were carried out under light irradiation. An NZT‐1 film (prepared by drop‐casting, see Supporting Information for details) reached 118.7°C in air (Figure 5e), significantly higher than a pristine TP‐TDS COF film (95.8°C, Figure S43). To mimic the actual reaction environment, we also measured powder samples dispersed in water. Under identical irradiation, NZT‐1 powder reached 70.9°C, while TP‐TDS powder reached 63.4°C (Figure S44). Thus, in both configurations (film in air or powder in water), NZT‐1 shows a stronger photothermal effect than the pristine COF. The lower absolute temperatures of the powder samples are due to efficient heat dissipation by the surrounding water. Control experiments (Figure 5f) further decouple the roles of light and heat: no H2 evolution occurs in the dark even at 80°C, but under illumination, raising the temperature from 25°C to 80°C monotonically boosts the rate from 439.8 to 517.7 mmol g^−1^ h^−1^, suggesting a genuine photo‐thermal synergy. These multiscale insights converge into a coherent photocatalytic pathway for NZT‐1 (Figure 5h): (i) light absorption/exciton generation in the COF; (ii) IEF‐driven charge separation and electron transfer to NiZnCo‐NC; (iii) proton reduction at the metal sites, aided by photothermally lowered desorption barriers; and (iv) efficient hole scavenging. The hierarchical integration of directed charge management and localized photothermal heating establishes a robust platform for high‐efficiency solar energy conversion.

**FIGURE 5:**
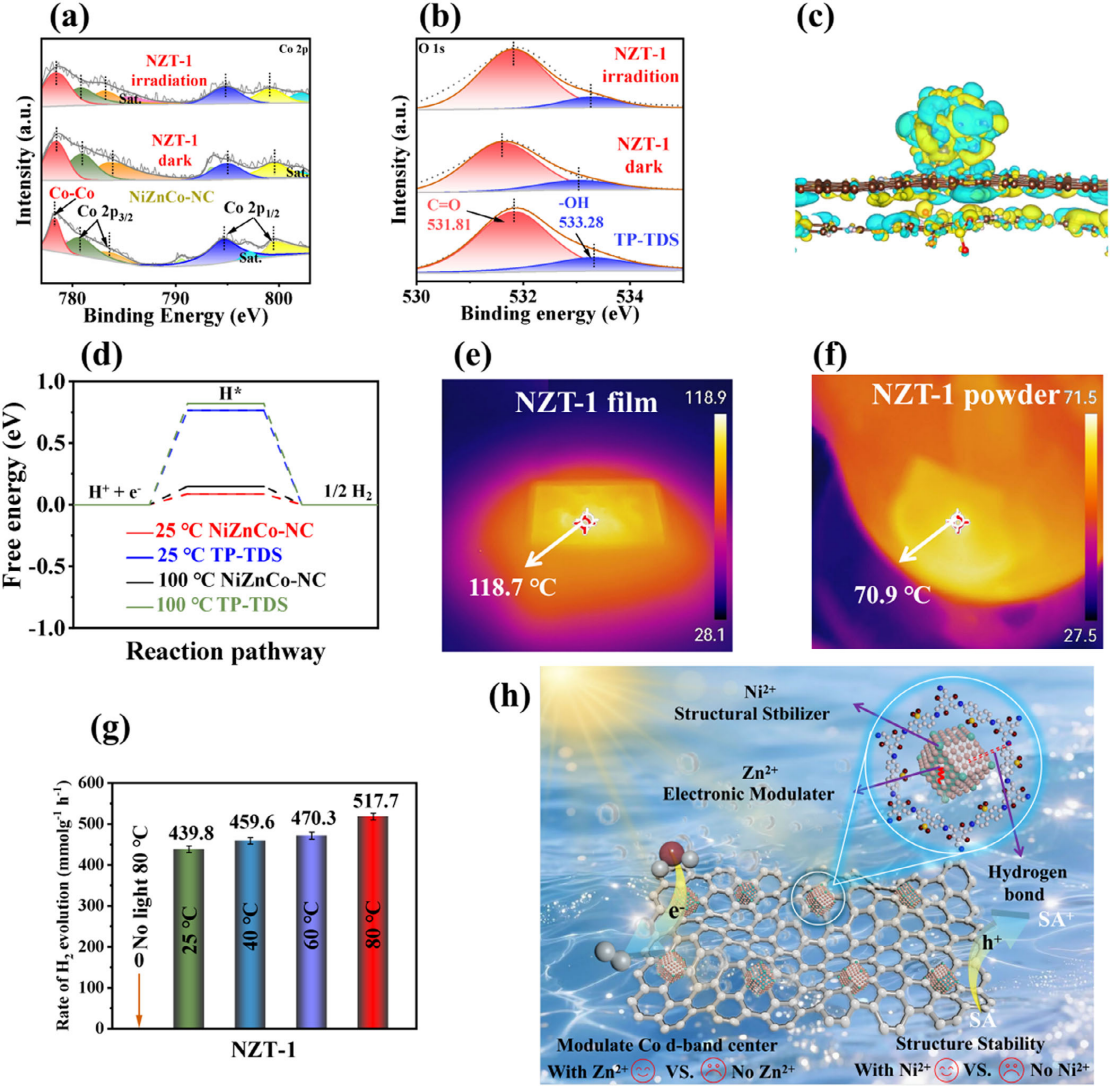
XPS and charge distribution analysis unveiling interfacial electron transfer and photothermal behaviour in photocatalysts. (a) Co 2p and (b) O 1s high‐resolution XPS spectra; (c) Differential charge density distribution mapped across the NZT‐x; (d) Calculated Gibbs free energy profiles for HER on different temperature; Infrared thermography capturing surface temperature evolution in (e) NZT‐1 film (f) NZT‐1 powder;(g) Photocatalytic H_2_ evolution rates of different temperature; (h) Proposed reaction mechanism illustrating photocatalytic hydrogen evolution pathway in the NZT‐x hybrid system.

## 3 Conclusions

In conclusion, we have successfully developed a high‐performance, noble‐metal‐free hybrid photocatalyst (NZT‐1) through a rational hydrogen‐bonding‐directed assembly strategy. A comprehensive suite of characterizations combined with theoretical calculations elucidates a coherent structure‐activity‐performance relationship. The activity stems from a hierarchical integration of multiple synergies: (i) a synergistic division of roles within the ternary NiZnCo‐NC cocatalyst, where Ni ensures structural integrity and Zn acts as an electronic modulator to rectify the over‐binding of hydrogen on Co, thereby optimizing its intrinsic catalytic activity; (ii) directional interfacial charge transfer driven by a built‐in electric field, enabled by the precisely engineered hydrogen‐bond‐bridged heterojunction; and (iii) a pronounced photothermal effect that synergistically enhances reaction kinetics both macroscopically and at the atomic level. This work highlights the importance of interface engineering and multicomponent synergy in designing efficient photocatalytic systems. The proposed assembly strategy may be extended to other COF‐based hybrid architectures for solar‐to‐chemical energy conversion.

## Author Contributions

**Ailing Pan**: Methodology, investigation, data curation, Writing – original draft. **De Yan**, investigation. **Anwu Xu**: Methodology, investigation, data curation. **Hong Du**: conceptualization, writing – review & editing, supervision. **Abulikemu Abudu Rexit**: Methodology. **Gang Zhang**: Theoretical calculation, investigation, data analysis.

## Conflicts of Interest

The authors declare no conflicts of interest.

## Supporting information




**Supporting File 1**: advs76700‐sup‐0001‐SuppMat.docx.

**Supporting File 2**: advs76700‐sup‐0002‐VideoS1.mp4.

## Data Availability

The data that support the findings of this study are available on request from the corresponding author. The data are not publicly available due to privacy or ethical restrictions.
