# Supplementary material for: Hydrogen‐Bonding‐Directed Assembly of COF/Nanocluster Hybrids With Synergistic Roles for Efficient Solar Hydrogen Production
Source: Adv Sci (Weinh). 2026 Jul 27;13(57):e76700. doi: 10.1002/advs.76700 (PMC13403373; doi:10.1002/advs.76700)
Supplement: Supplementary file 1 [file ADVS-13-e76700-s001.docx]

Hydrogen-Bonding-Directed Assembly of COF/Nanocluster Hybrids with Synergistic Roles for Efficient Solar Hydrogen Production

*Author(s): Ailing Pan,^a^ De Yan,^#, a^ Anwu Xu,^d^* *Hong Du,^*a,b^ Abulikemu Abudu Rexit,^a^ Gang Zhang^*c^*

^a.^ College of Chemistry and Chemical Engineering, Xinjiang Normal University, Urumqi, 830054, China.

^b.^Xinjiang Key Laboratory of Energy Storage and Photoelectrocatalytic Materials, Urumqi, 830054, China

^c.^Xinjiang Joinworld Co., Ltd, Urumqi 830091, PR China.

^d.^ Department of Chemistry, University of Science and Technology of China, Hefei, Anhui 230026, P.R. China

^† #^Contributed equally to this work.

*Corresponding Author:175790509@ qq.com (Prof. Hong Du); 1498042333@qq.com (Prof. Gang Zhang)

**Materials and Methods**

**Materials**

All chemicals were used as received without further purification. 1,4-Dioxane, mesitylene, tetrahydrofuran (THF), acetone, ascorbic acid, anhydrous sodium sulfate (Na_2_SO_4_), cobalt(II) nitrate hexahydrate (Co(NO_3_)_2_), nickel(II) nitrate hexahydrate (Ni(NO_3_)_2_), zinc(II) nitrate hexahydrate (Zn(NO_3_)_2_), 2-methylimidazole (2-MIM), methanol, sulfuric acid (H_2_SO_4_, 0.5 M aqueous solution was used), and glacial acetic acid were purchased from Tianjin Zhiyuan Chemical Reagent Co., Ltd. 2,4,6-Triformylphloroglucinol (TP) and 3,7-diaminodibenzo [b,d] thiophene-5,5-dioxide (TDS) were obtained from Shanghai Aladdin Biochemical Technology Co., Ltd.

**Synthesis of ZIF-67-based Precursors (M-ZIF-67)**

A series of bimetallic and trimetallic ZIF-67 precursors were synthesized via a modified co-precipitation method at ambient temperature. The general procedure for NiZnCo-ZIF-67 is as follows: Solution A was prepared by dissolving Co(NO_3_)_2_ (0.5821 g, 3.2 mmol), Ni(NO_3_)_2_ (0.073 g, 0.4 mmol), and Zn(NO_3_)_2_ (0.0756 g, 0.4 mmol) in methanol (50 mL). Solution B was prepared by dissolving 2-methylimidazole (2-MIM, 1.6222 g, 19.8 mmol) in methanol (50 mL). Under vigorous stirring, Solution A was rapidly poured into Solution B. The mixture was stirred for 1 hour and then aged at room temperature for 24 hours. The resulting dark-purple precipitate was collected by centrifugation, washed thoroughly with methanol three times, and dried at 60 °C overnight.

Control precursors with different metal compositions were synthesized by adjusting the metal salts in Solution A while keeping the total molar quantity of metal ions (2.5 mmol) and the 2-MIM quantity constant. Co-ZIF-67 was prepared using only Co(NO_3_)_2_ (0.73 g, 4 mmol). NiCo-ZIF-67 was prepared using Ni(NO_3_)_2_ (0.1454 g, 0.8 mmol) and Co(NO_3_)_2_ (0.5821 g, 3.2 mmol). ZnCo-ZIF-67 was prepared using Zn(NO_3_)_2_ (0.1512 g, 0.8 mmol) and Co(NO_3_)_2_ (0.5821 g,3.2 mmol).

**Synthesis of N-doped Carbon Encapsulated Metal Nanoclusters (M-NC)**

The as-synthesized ZIF-67 precursors were converted into conductive N-doped carbon matrices via pyrolysis. Taking NiZnCo-ZIF-67 as an example: The precursor powder was placed in a quartz boat and annealed in a tube furnace under a continuous Ar flow. The temperature was raised to 900 °C at a rate of 5 °C min⁻^1^, held for 3 hours, and then allowed to cool naturally to room temperature. The obtained black powder was then stirred in 0.5 M H_2_SO_4_ aqueous solution for 24 hours to remove unstable metal species and acid-leachable impurities. The product was collected by centrifugation, washed with deionized water until the supernatant reached neutral pH, and finally dried at 60 °C to obtain the final product, denoted as NiZnCo-NC. The control samples Co-NC, NiCo-NC, and ZnCo-NC were synthesized under identical pyrolysis and acid-washing conditions using their corresponding ZIF-67 precursors (Co-ZIF-67, NiCo-ZIF-67, ZnCo-ZIF-67).

**Synthesis of TP-TDS COF and Hybrid Catalysts**

The TP-TDS COF and its integrated hybrid catalysts with M-NC substrates were synthesized via a solvothermal Schiff-base condensation. The synthesis of the optimized hybrid catalyst NZT-1 is described as the representative procedure. In a typical synthesis targeting a 1:1 mass ratio of the NiZnCo-NC component to the total organic monomers (TP + TDS), NiZnCo-NC (46.4 mg) and TDS (29.6 mg) were first dispersed in 1,4-dioxane (1.5 mL) and stirred for 30 minutes to allow pre-adsorption. Subsequently, TP (16.8 mg) and mesitylene (1.5 mL) were added. After ultrasonication for 10 minutes, aqueous acetic acid (0.3 mL, 6 M) was introduced as a catalyst. The mixture was purged with N_2_ for 20 minutes, sealed, and heated at 120 °C in an oil bath for 72 hours. The resulting product was collected by centrifugation, washed alternately with acetone and THF (three times each) to remove unreacted monomers and oligomers. The product was then purified by alternate soaking in fresh THF and acetone (5 hours per solvent) over a period of 24 hours, followed by drying under vacuum at 60 °C for 12 hours. The pure TP-TDS COF was obtained following an identical protocol but in the absence of any M-NC particles, with the monomers TP (16.8 mg) and TDS (29.6 mg) directly dispersed in a 1:1 (v/v) mixed solvent of mesitylene and 1,4-dioxane (1.5 mL each).

Variants of the hybrid catalyst with different NiZnCo-NC loadings (denoted as NZT-0.5, NZT-2, and NZT-3) were prepared by the same procedure, adjusting the mass of NiZnCo-NC to achieve nominal NC-to-monomers mass ratios of 1:0.5, 1:2, and 1:3, respectively. Control hybrid catalysts, where the NiZnCo-NC was replaced with an equivalent mass (46.4 mg) of Co-NC, NiCo-NC, or ZnCo-NC, were synthesized under identical conditions to yield samples CT-1, NCT-1, and ZCT-1.

For the critical control experiment, a physically mixed sample was prepared by manually grinding pre-synthesized NiZnCo-NC (46.4 mg) and TP-TDS COF together in an agate mortar for 20 minutes. This sample is denoted as milled NZT-1.

Scheme S1 Schematic illustration of the synthetic procedures for TP-TDS COF

**Preparation of NZT-1 and TP-TDS COF film for photothermal measurement**

10 mg of NZT-1 catalyst was dispersed in a mixed solvent containing 1 mL of ethanol and ethylene glycol (1:1, v/v), followed by the addition of 80 μL of Nafion solution (5 wt%) as a binder. The mixture was ultrasonicated for 30 min to obtain a homogeneous suspension. The suspension was then drop‑cast onto a 2 × 3 cm FTO glass substrate and dried at 60 °C for 12 h. To achieve a uniform film covering the entire substrate, the drop‑casting and drying process was repeated three times. The same procedure was used to prepare the TP-TDS COF film as a control.

**Materials Characterization**

The physicochemical properties of all synthesized materials were systematically characterized using a suite of complementary techniques. Crystalline structures were analyzed by X-ray diffraction (XRD, Bruker D8 Advance) with Cu Kα radiation (λ = 1.5406 Å), collecting data in the 2θ range of 2° to 80° at a scanning rate of 5° min⁻^1^. Chemical functional groups were identified via Fourier transform infrared spectroscopy (FT-IR, Thermo Scientific Nicolet iS10) in transmission mode with KBr pellets. Morphological and microstructural information was obtained using scanning electron microscopy (SEM, ZEISS Sigma 300) and high-resolution transmission electron microscopy (HRTEM, FEI Talos F200x). The HRTEM system was equipped with an energy-dispersive X-ray spectroscopy (EDS) detector for elemental mapping. Surface chemical states and elemental compositions were analyzed by X-ray photoelectron spectroscopy (XPS, Thermo Scientific K-Alpha), with binding energies calibrated to the C 1s peak at 284.8 eV. Optical absorption properties were assessed by UV-visible diffuse reflectance spectroscopy (UV-vis DRS, Shimadzu UV-3600) using BaSO_4_ as a reflectance standard. Textural properties, including specific surface area and pore size distribution, were determined from N₂ adsorption-desorption isotherms measured at 77 K (Micromeritics ASAP 2460). Specific surface areas were calculated via the Brunauer-Emmett-Teller (BET) method, and pore size distributions were derived from the adsorption branch using the Barrett-Joyner-Halenda (BJH) model. Inductively coupled plasma optical emission spectroscopy (ICP-OES, Agilent 5110) was employed to quantify the metal content. Charge carrier dynamics were studied by time-resolved photoluminescence spectroscopy (Edinburgh Instruments FLS1000, excitation at 375 nm). The local atomic coordination environment of metallic centers was further investigated using X-ray absorption fine structure (XAFS) spectroscopy at the corresponding metal K-edges.

**Photocatalytic Hydrogen Evolution**

The photocatalytic hydrogen evolution reaction (HER) was evaluated in a sealed quartz reactor. Typically, the photocatalyst (2 mg) was dispersed in an aqueous solution (45 mL) containing ascorbic acid (0.1 M) as a sacrificial donor. The suspension was ultrasonicated for 20 minutes to ensure homogeneity and then purged with high-purity N_2_ for 30 minutes to remove dissolved oxygen. The reaction mixture was irradiated using a 300 W Xe lamp equipped with an AM 1.5G filter and a 420 nm cutoff filter to simulate visible-light irradiation (λ > 420 nm). The light intensity at the sample surface was adjusted to 100 mW cm⁻^2^ using a calibrated power meter. The evolved hydrogen gas was quantified at regular intervals via an online gas chromatograph (GC 7000, TCD) equipped with a molecular sieve column and using N_2_ as the carrier gas.

**Photoelectrochemical (PEC) Measurements**

PEC properties were characterized using a standard three-electrode system connected to an electrochemical workstation (CHI 660). A Pt wire and an Ag/AgCl (saturated KCl) electrode served as the counter and reference electrodes, respectively. The working electrode was prepared by drop-casting a catalyst ink onto a fluorinated tin oxide (FTO) glass substrate (1 × 1 cm^2^). The ink was formulated by dispersing the catalyst (5 mg) in a mixture of ethanol (0.25 mL), ethylene glycol (0.25 mL), and Nafion solution (80 µL, 5 wt%), followed by sonication for 30 minutes. The coated FTO glass was dried at 60 °C for 12 hours. Measurements were conducted in an aqueous Na_2_SO_4_ electrolyte (0.5 M) under chopped illumination from a 300 W Xe lamp.

**Computational Methods**

We have employed the first-principles tool-Vienna Ab initio Simulation Package(VASP) ^[1,2]^ to perform all density functional theory (DFT) calculations within the generalized gradient approximation (GGA) using the Perdew-Burke-Ernzerhof (PBE) ^[3]^ formulation. We have chosen the projected augmented wave (PAW) potentials ^[4, 5]^ to describe the ionic cores and take valence electrons into account using a plane wave basis set with a kinetic energy cutoff of 450 eV. Partial occupancies of the Kohn−Sham orbitals were allowed using the Gaussian smearing method and a width of 0.05 eV. For the optimization of both geometry and lattice size, the Brillouin zone integration was performed with 1×1×1 *Γ*-centered *k*-point sampling .^[6]^ The self-consistent calculations applied a convergence energy threshold of 10^-5^ eV. The equilibrium geometries and lattice constants were optimized with maximum stress on each atom within 0.02 eV Å^-1^. The 15 Å vacuum layer was normally added to the surface to eliminate the artificial interactions between periodic images. The weak interaction was described by DFT+D3 method using empirical correction in Grimme’s scheme.^[7-9]^ Spin polarization method was adopted to describe the magnetic system. The Gibbs free energy for each elementary step was calculated as: G = E_elec_ + E_ZPE_ – TS, in which E_elec_ is the electronic energy at 0 K calculated by DFT, E_ZPE_ is the zero-point energy term, and T is the absolute temperature (here 298 K), S is the entropy. We note that the DFT models used here are idealized (e.g., periodic slabs, vacuum conditions, 0 K) and do not fully account for complex catalytic environments such as solvent effects, defects, or dynamic restructuring. Therefore, the calculated trends should be interpreted as supportive evidence rather than definitive proof.

**Statistical Analysis**

All experimental data were obtained from at least three independent replicates to ensure reproducibility. Data for photocatalytic HER are presented as the mean value with error bars representing the standard deviation. The raw hydrogen evolution data were normalized to the mass of the catalyst. For XPS analysis, binding energies were uniformly calibrated to the C 1s peak at 284.8 eV. No data points were excluded from the analysis. Statistical analysis and graph preparation were performed using Origin 2022 software.

**Fig. S1** XRD patterns of NiZnCo-ZIF-67

**Fig. S2** XRD patterns of NiZnCo-NC

**Fig. S3** XRD patterns of TP-TDS COF and NZT-1

**Fig. S4** Comparison of samples before and after acid treatment. Photographs showing the distinct color of pristine NiZnCo-ZIF-67, its immediate change upon H_2_SO_4_ addition, and the resulting NiZnCo-NC material.

Control experiment against unwashed precursors: We designed a dedicated acid-wasting stability test to address the possibility of unwashed precursors. It is important to highlight that the NiZnCo-NC catalyst had already undergone standard purification with 0.5 M H_2_SO_4_ during its synthesis to remove unstable species. The newly designed stability test confirmed that although the pristine NiZnCo-ZIF-67 precursor dissolves immediately in acid, the final NiZnCo-NC catalyst remains completely stable under the same harsh conditions (0.5 M H_2_SO_4_, 24 h). This result clearly demonstrates that the detected Ni and Zn are structurally incorporated components rather than residual precursors.

**Fig. S5** HRTEM image of the (a, b) TP-TDS COF; (c, d) NZT-1.

HRTEM analysis also provides insight into the structural evolution of the COF component before and after hybridization. Pristine TP-TDS COF exhibits discernible locally ordered lattice fringes, consistent with its crystalline nature revealed by XRD. After hybridization with NiZnCo-NC, the COF domains in NZT-1 display less distinct lattice fringes, suggesting partial disruption of long-range ordering during the interfacial assembly process. Nevertheless, the characteristic diffraction peak of TP-TDS COF remains observable in the XRD pattern of NZT-1, although with reduced intensity, indicating that the COF framework is retained rather than completely amorphized after hybridization. Therefore, the decreased lattice ordering observed by HRTEM is attributed to reduced stacking regularity and local structural distortion induced by the confined growth of COF around NiZnCo-NC nanoclusters. Importantly, this partial loss of long-range order is accompanied by exfoliation of the COF into ultrathin nanosheets (Fig. 2b-c and Fig. S10), which enhances active site accessibility and overall photocatalytic performance.

**Table S1** The ICP-OES of NiZnCo-ZF-67 and NiCoZn-NC samples

| Samples | NiZnCo-ZIF-67 | NiZnCo-NC |
| --- | --- | --- |
| Co content (wt%) | 22.37 | 14.39 |
| Ni content (wt%) | 0.089 | 0.025 |
| Zn content (wt%) | 2.49 | 0.055 |

**Fig. S6** EDS spectrum of the NZT-1 catalyst;

**Fig. S7** ^1^NMR of TP-TDS.

**Fig. S8** ^1^NMR of NiZnCo-NC.

**Fig. S9** ^1^NMR of NZT-1.

**Fig. S10** Photocatalytic H_2_ evolution rates of milled NZT-1 and NZT-1under AM 1.5G.

**Fig. S11** Multiple AFM high sensor profile of the (a) TP-TDS and (c) NZT-1. Thickness distribution histogram of the (b) TP-TDS and (d ) NZT-1

**Fig. S12** H_2_ evolution rate of samples

**Fig. S13** H_2_ evolution rate of samples under (a) AM 1.5G and (b) visible light (λ ≥ 420 nm) irradiation;

**Fig. S14** AQY of NZT-1.

**Table S2** The AQY of NZT-1 heterojunction

| Wavelength  (nm) | 420 | 500 | 550 | 600 | 650 |
| --- | --- | --- | --- | --- | --- |
| H_2_ evolution  (μmol) | 10.04 | 478 | 0.94 | 0.20 | 0.28 |
| Light intensity  (mW/cm^2^) | 61.00 | 85.93 | 97.50 | 90.93 | 40.31 |
| Irradition area  (cm^2^) | 4 | 4 | 4 | 4 | 4 |
| Irradition time  (h) | 1 | 1 | 1 | 1 | 1 |
| AQY(%) | 0.65 | 18.6 | 0.029 | 0.006 | 0.018 |

**^a^** Condition: Ascorbic acid (0.1 M, 45 mL) as sacrificial agent; 300 W Xe lamp equipped with various band-pass filters as light source;

λ=500 nm:

The number of incident photons:

N = $\frac{\text{Eλ}}{\text{hc}}$ = $\frac{\text{85.93}\text{×}\text{4}\text{×}\text{10}^{\text{−3}}\text{×}\text{1}\text{×}\text{3600}\text{×}\text{500}\text{×}\text{10}^{\text{−9}}}{\text{6.626}\text{×}\text{10}^{\text{−34 }}\text{×}\text{3}\text{×}\text{10}^{\text{8}}}\text{ }$= 3.1$\text{×}\text{10}^{\text{21}}$

AQY:

AQY = $\frac{\text{2 }\text{×}\text{ }\text{t}\text{h}\text{e}\text{ }\text{number}\text{ }\text{of}\text{ }\text{evolved}\text{ }\text{H}_{\text{2}}\text{ }\text{molecules}}{\text{N}}$ $\text{×}\text{100\%}$

=$\frac{\text{2 }\text{×}\text{ 6.02 }\text{×}\text{ }\text{10}^{\text{23 }}\text{×}\text{478}\text{×}\text{ }\text{10}^{\text{−6}}}{\text{3.1}\text{×}\text{10}^{\text{21}}}\text{×}\text{1}$*00%*

=18.6%

**Table S3** The AQY of NZT-1 heterojunction under different light intensity.

| Wavelength  (nm) | 500 | 500 | 500 |
| --- | --- | --- | --- |
| H_2_ evolution  (μmol) | 201 | 315 | 478 |
| Light intensity  (mW/cm^2^) | 40.13 | 60.28 | 85.93 |
| Irradition area  (cm^2^) | 4 | 4 | 4 |
| Irradition time  (h) | 1 | 1 | 1 |
| AQY(%) | 16.7 | 17.4 | 18.6 |

The number of incident photons:

N = $\frac{\text{Eλ}}{\text{hc}}$ = $\frac{\text{40.13}\text{×}\text{4}\text{×}\text{10}^{\text{−3}}\text{×}\text{1}\text{×}\text{3600}\text{×}\text{500}\text{×}\text{10}^{\text{−9}}}{\text{6.626}\text{×}\text{10}^{\text{−34 }}\text{×}\text{3}\text{×}\text{10}^{\text{8}}}\text{ }$= 1.45$\text{×}\text{10}^{\text{21}}$

AQY:

AQY = $\frac{\text{2 }\text{×}\text{ }\text{t}\text{h}\text{e}\text{ }\text{number}\text{ }\text{of}\text{ }\text{evolved}\text{ }\text{H}_{\text{2}}\text{ }\text{molecules}}{\text{N}}$ $\text{×}\text{100\%}$

=$\frac{\text{2 }\text{×}\text{ 6.02 }\text{×}\text{ }\text{10}^{\text{23 }}\text{×}\text{201}\text{×}\text{ }\text{10}^{\text{−6}}}{\text{1.45}\text{×}\text{10}^{\text{21}}}\text{×}\text{1}$*00%*

=16.7%

Solar-to-chemical conversion efficiency(SCC)：

SCC(%)=$\frac{r_{H_{2}\times\Delta G}}{P_{Light}\times A}$

where:

$\text{r}_{\text{H}_{\text{2}}}$ = hydrogen evolution rate in mol s⁻^1^

ΔG = 237 kJ mol⁻^1^ (Gibbs free energy for H₂ production)

P_light_ = incident light intensity (100 mW cm⁻^2^ = 0.1 W cm⁻^2^)

A = illuminated area (4 cm^2^)

From the experimental data:

H_2_ evolution rate = 439.8 mmol g⁻^1^ h⁻^1^

Catalyst amount = 2 mg = 0.002 g

$r_{H_{2}}$ = $\frac{8.796 \times{10}^{-4}}{3600}$ = 2.443 $\times$ 10^-7^ mol s^-1^

Thus: SCC(%) = $\frac{r_{H_{2}\times\Delta G}}{P_{Light}\times A}$ = $\frac{2.443 \times{10}^{-7} \times237000}{0.1 \times4}$ $\times$ 100% = 14.5%

**Fig. S15** (a) H_2_ evolution rate of different samples under λ ≥ 420 nm;

**Fig. S16** Comparisons of photocatalytic hydrogen production properties of the reported NZT-1^[10-19]^

**Fig. S17** Water contact angle measurements of different samples. Contact angle images of TP-TDS COF recorded during the 1 s (a) and 2 s (b) measurements, NiZnCo-NC during the 1 s (c) and 2 s (d) measurements, and NZT-1 during the 1 s (e) and 2 s (f) measurements.

**Fig. S18** (a) XRD of before and after the reaction NZT-1

**Fig. S19** SEM of (a) before and (b) after the reaction NZT-1

**Fig. S20** TEM of (a) before and (b) after the reaction NZT-1

**Fig. S21** XPS of before and after the reaction NZT-1

**Fig. S22** SEM of (a) Co-NC; (b) NiCo-NC; (c) ZnCo-NC; (d) NiZnCo-NC.

The additional SEM images in Fig. S15a-d provide direct and striking visual evidence of the distinct structural roles played by Ni and Zn. The control sample Co-NC (Fig. S15a) partially maintains the ZIF-67 dodecahedron morphology but exhibits significant structural collapse in certain areas. Critically, the introduction of Ni in NiCo-NC (Fig. S15b) results in a well-preserved dodecahedral structure with no apparent collapse, demonstrating that Ni doping is essential for achieving robust architectural stability. In stark contrast, ZnCo-NC (Fig. S15c) completely loses the dodecahedral framework, indicating that Zn incorporation severely disrupts the structural integrity. Most importantly, the ternary NiZnCo-NC (Fig. S15d) successfully maintains the intact dodecahedral morphology, unambiguously proving that Ni is the key element responsible for stabilizing the framework, effectively counteracting the destabilizing effect of Zn. This evidence definitively proves that Ni is a structurally integrated and crucial component for maintaining the catalyst’s integrity.

**Fig. S23** The substrate structure of Co-NC.

**Fig. S24** The substrate structure of NiCo-NC.

**Fig. S25** The substrate structure of ZnCo-NC.

**Fig. S26** The substrate structure of NiZnCo-NC.

**Fig. S27** The absorption H structure of Co-NC.

**Fig. S28** The absorption H structure of NiCo-NC.

**Fig. S29** The absorption H structure of ZnCo-NC.

**Fig. S30** The absorption H structure of NiZnCo-NC.

**Fig. S31** The structure of TP-TDS.

**Fig. S32** The absorption H structure of TP-TDS.

**Fig.S33** E_g_ of dendrite-mimetic TP-TDS COF

Based to the Kubelka-Munk function plot switch from the corresponding UV-vis, the band gap value of dendrite-mimetic TP-TDS COF is calculated to be 2.08 eV.

**Fig. S34** wavelet transform (WT) contour plots of the k^2^ -weighted Co K-edge EXAFS of (a) NZT-1, (b) Co-foil, (c) Co-Pc, (d) CoO, (e) Co_2_O_3_, (f) Co_3_O_4_, respectively.

**Fig. S35** (a) K-space of (a) Co-foil and (b) NZT-1 samples.

**Fig. S36** R-space of (a) Co-foil and (b) NZT-1 samples.

**Table S4** EXAFS fitting parameters at the Co K-edge for various samples（Ѕ_0_^2^=0.70from Co-foil）

|  | shell | CN*^a^* | R*^b^*(Å) | σ^2^*^c^*(Å^2^) | ΔE_0_*^d^*(eV) | R factor |
| --- | --- | --- | --- | --- | --- | --- |
| Co foil | Co-Co | 12 | 2.49±0.01 | 0.0063 | 6.93±0.3 | 0.0008 |
| NZT-1 | Co-N | 1.93±0.32 | 2.00±0.01 | 0.0098 | 6.79±0.8 | 0.0034 |
|  | Co-Co | 6.46±0.17 | 2.49±0.02 | 0.0063 |  |  |

*^a^CN*: coordination numbers; *^b^R*: bond distance; *^c^σ*^2^: Debye-Waller factors; *^d^* Δ*E*_0_: the inner potential correction. R factor: goodness of fit. Error bounds that characterize the structural parameters obtained by EXAFS spectroscopy were estimated as CN±20%; R ± 1%; σ^2^ ± 20%.

**Fig. S37** Electrochemical impedance spectroscopy (EIS) Nyquist plots of TP-TDS COF (red) and NZT-1 (blue).

**Fig.S38** Pore size of TP-TDS COF, NiZnCo-NC, and NZT-1

**Table S5** BET surface area, Pore volume, average pore size of TP-TDS COF, NiZnCo-NC and NZT-1

| Samples | BET surface area  (cm^2^ g^-1^) | Pore Volume  (cm^3^ g^-1^) | Average pore size (nm) |
| --- | --- | --- | --- |
| TP-TDS | 986.5 | 0.56 | 9.97 nm |
| NiZnCo-NC | 98.28 | 0.22 | 9.87 nm |
| NZT-1 | 410.83 | 0.32 | 9.43 nm |

**Fig. S39** UPS spectra of (a) TP-TDS and (b) NiZnCo-NC

**Fig. S40** XPS spectra of C 1s

**Fig. S41** XPS spectra of (a) N 1s; (b) S 2p.

**Fig. S42** XPS spectra of N 1s;

**Fig. S43** Infrared thermography capturing surface temperature in TP-TDS film.

**Fig. S44** Infrared thermography capturing surface temperature in TP-TDS power.

**Reference:**

1. Kresse, G.; Furthmüller, J. Efficiency of ab-initio total energy calculations for metals and semiconductors using a plane-wave basis Set. Comput. Mater. Sci. 1996, 6, 15−50.
2. Kresse, G.; Furthmüller, J. Efficient iterative schemes for ab initio total-energy calculations using a plane-wave basis set. Phys. Rev. B 1996, 54, 11169−11186.
3. Perdew, J. P.; Burke, K.; Ernzerhof, M. Generalized gradient approximation made simple. Phys. Rev. Lett. 1996, 77, 3865−3868.
4. Kresse, G.; Joubert, D. From ultrasoft pseudopotentials to the projector augmented-wave method. Phys. Rev. B 1999, 59, 1758-1775.
5. Blöchl, P. E. Projector augmented-wave method. Phys. Rev. B 1994, 50, 17953-17979.
6. Monkhorst H J, Pack J D. Special points for brillouin-zone integrations[J]. Phys. Rev. B, 1976, 13(12): 5188.
7. [S. Grimme, J. Antony, S. Ehrlich, and S. Krieg, J. Chem. Norm-conserving pseudopotentials with chemical accuracy compared to all-electron calculations. Phys. 2010，132, 154104.](http://aip.scitation.org/doi/full/10.1063/1.3382344)
8. [S. Grimme, S. Ehrlich, and L. Goerigk, Effect of the damping function in dispersion corrected density functional theory J. Comp. Chem. 2011，32, 1456.](http://onlinelibrary.wiley.com/doi/10.1002/jcc.21759/abstract)
9. Dong W, Yu X, Qin Z, Chen Y, Ren S, Li L. Synergistic enhancement of photocatalytic hydrogen evolution in covalent organic frameworks via isoreticular design, isomerism, and protonation. *Angewandte Chemie International Edition* 2025.
10. Du X, Ji H, Xu Y, Du S, Feng Z, Dong B*, et al.* Covalent organic framework without cocatalyst loading for efficient photocatalytic sacrificial hydrogen production from water. *Nature Communications* 2025, **16**(1).
11. Gao R, Shen R, Huang C, Huang K, Liang G, Zhang P*, et al.* 2D/2D hydrogen‐bonded organic frameworks/covalent organic frameworks S‐scheme heterojunctions for photocatalytic hydrogen evolution. *Angewandte Chemie International Edition* 2024, **64**(2).
12. Khalil IE, Das P, Küçükkeçeci H, Dippold V, Rabeah J, Tahir W*, et al.* Hierarchical porous covalent organic frameworks: The Influence of additional macropores on photocatalytic hydrogen evolution and hydrogen peroxide production. *Chemistry of Materials* 2024, **36**(17)**:** 8330-8337.
13. Li H, Fan J, Ran M, Borse RA, Lin SX, Yuan D. Design and construction of D‐A‐extended 3D covalent–organic frameworks for boosting photocatalytic hydrogen evolution. *Angewandte Chemie International Edition* 2025, **64**(24).
14. Lin Z, Yu X, Zhao Z, Ding N, Wang C, Hu K*, et al.* Controlling crystallization in covalent organic frameworks to facilitate photocatalytic hydrogen production. *Nature Communications* 2025, **16**(1).
15. Ma S, Li Z, Hou Y, Li J, Zhang Z, Deng T*, et al.* Fully conjugated benzobisoxazole‐bridged covalent organic frameworks for boosting photocatalytic hydrogen evolution. *Angewandte Chemie International Edition* 2025, **64**(19).
16. Shen R, Huang C, Hao L, Liang G, Zhang P, Yue Q*, et al.* Ground-state charge transfer in single-molecule junctions covalent organic frameworks for boosting photocatalytic hydrogen evolution. *Nature Communications* 2025, **16**(1).
17. Shen R, Huang C, Hao L, Liang G, Zhang P, Yue Q*, et al.* Ground-state charge transfer in single-molecule junctions covalent organic frameworks for boosting photocatalytic hydrogen evolution. *Nature Communications* 2025, **16**(1).
18. Wang T, Li M, Chen Y, Che X, Bi F, Yang Y*, et al.* Regioisomeric benzotriazole-based covalent organic frameworks for high photocatalytic activity. *ACS Catalysis* 2023, **13**(23)**:** 15439-15447.
19. Zhao W, Luo L, Cong M, Liu X, Zhang Z, Bahri M*, et al.* Nanoscale covalent organic frameworks for enhanced photocatalytic hydrogen production. *Nature Communications* 2024, **15**(1).
